# Editorial: Neurological and clinical aspects of perinatal mental health

**DOI:** 10.3389/fpsyt.2023.1188080

**Published:** 2023-04-11

**Authors:** Tom Kingstone, Karen M. Tabb, Yuan-Pang Wang

**Affiliations:** ^1^School of Medicine, Keele University, Staffordshire, United Kingdom; ^2^Beckman Institute, University of Illinois Urbana-Champaign, Champaign, IL, United States; ^3^Institute and Department of Psychiatry (LIM-23), School of Medicine, University of São Paulo, São Paulo, Brazil

**Keywords:** perinatal mental health, women, maternal, neurological, clinical

## Background

Perinatal mental health refers to a variety of experiences, disorders, and diagnoses experienced by women during pregnancy and up to 12 months after birth. During the perinatal period, around 1-in-5 women experience a mental health problem, such as an anxiety disorder (e.g., Generalized Anxiety Disorder, Obsessive-Compulsive Disorder), depression, or an episode of psychosis—estimates vary depending on the type of mental health problem and biological, psychological, and social factors (1). It is clear from existing research that early identification of these problems is important to avoid the exacerbation of symptoms and improve long term outcomes for both mother and baby (2).

In the context of COVID-19, emerging evidence indicates an increase in the incidence rates for perinatal mental health problems during the pandemic (3). Such reported increases during COVID-19 exert further pressure on families and health and social care services. These increased burdens, coupled with gaps in our knowledge, highlight the essential need for up-to-date research to inform clinical practice and treatment.

The Research Topic “*Neurological and clinical aspects of perinatal mental health*” includes eight articles covering a range of perspectives, study methodologies and contexts. Each article makes a novel contribution to extend the evidence-base for perinatal mental health. Four overarching themes are covered:

- Epidemology of perinatal mental health (Faisal-Cury et al.)
- Neurobiological aspects of perinatal mental health (Cheng et al.; Mao et al.)
- Screening of perinatal mental health problems (Koukopoulos et al.)
- Non-pharmacological interventions and services to improve perinatal mental health outcomes (Desai et al.; Kornfield et al.; Segre et al.; Hicks et al.)

## Epidemiology: Suicide ideation and perinatal depression

Suicide is the leading cause of maternal mortality during the perinatal period (4). Suicidal ideation, a key precursor to suicidal death, with prevalence rates ranging from 3 to 30% (5). Evidence for an association between suicidal ideation and depression seems unclear among women during pregnancy (Faisal-Cury et al.). To address this gap in evidence, Faisal-Cury et al. took applied an epidemiological methodology to examine the role of depression in moderating suicidal ideation between pregnant and non-pregnant women in a Brazilian context. The authors describe important implications for clinical practice through and the identification of risk factors for suicide ideation, including a recent diagnosis of clinical depression.

## Neurobiological aspects of perinatal mental health

Substantive progress has been made in recent years to understand key aspects of the neurobiology of maternal mental illness, such as, neuroplasticity and neuroendocrine and immune system changes (6). However, further research is needed to understand the neurobiological mechanisms underlying specific perinatal mental health disorders, such as antenatal depression. Cheng et al. conducted a voxel-based whole-brain analysis of 43 singleton parents to elucidate the neurobiological features of antenatal depression during the COVID-19 pandemic. Mao et al. examined potential biomarkers for antenatal depression through tandem mass spectrometry methods. The authors aimed to establish potential for a serum metabonomic method in the early diagnosis of antenatal depression. Both primary research studies add to the evidence base for early detection of antenatal depression.

## Screening and assessment

There are few validated measures that have been designed specifically to screen for and assess the severity of perinatal anxiety. Validated measures in maternal mental health tend to focus on depression (e.g., Edinburgh Postnatal Depression Scale). Alternatively, standardized measures for anxiety disorders may be used as a proxy for perinatal anxiety [e.g., Generalized Anxiety Disorder-7 item (GAD-7)]. The Perinatal Anxiety Screening Scale (PASS) has been developed specifically to screen for anxiety across the perinatal period and include four dimensions: acute anxiety and adjustment, general worry and specific fears, perfectionism, control and trauma, and social anxiety (7). Koukopoulos et al. recognized that PASS had not been validated for use among Italian women; their article aimed to address and demonstrate the reliability and validity of PASS for this population.

## Non-pharmacological interventions

Pharmacological and non-pharmacological interventions for maternal mental health problems provide an important resource for women during the perinatal period. A recent review of perinatal anxiety management highlighted a range of non-pharmacological interventions such as, psychological therapies, mind-body interventions, supportive interventions and alternative therapies delivered—the strength of evidence varied (8). Further research is needed to better establish the evidence base for non-pharmacological interventions in terms of efficacy and effectiveness. Articles in this Research Topic describe three different non-pharmacological interventions, each at a different stage of development. Segre et al. seek to establish proof-of-concept of a telehealth listening visit intervention for emotionally distressed mothers of hospitalized newborns. Listening visits were nurse-delivered and involved reflective listening and problem-solving with mothers of hospitalized newborns. Kornfield et al. aimed to test the efficacy of a brief psychotherapeutic intervention for post-traumatic stress disorder (PTSD) in pregnant women. The authors present positive findings based on outcome data and recommend future research to measure effectiveness. Desai et al. reviewed the existing evidence from randomized controlled trials on the effectiveness of dietary supplements (probiotic, prebiotic, and symbiotic) to improve perinatal mental health in mothers. Each article (Segre et al.; Kornfield et al.; Desai et al.) identify promising areas for non-pharmacological interventions and recommendations for future research.

Hicks et al. offers a Canadian “view from the ground” on current clinical practice through their description of findings from a cross-sectional survey of perinatal mental health providers. The authors collate opinions from multiple stakeholders involved in perinatal mental health and describe important gaps in provision linked to training, screening, and management of perinatal mental health problems. This study highlights the need for researchers in perinatal mental health to plan effective dissemination strategies to support translation of research findings and engagement among practitioners.

## Conclusion

Articles in this Research Topic help to raise awareness about perinatal mental health and key issues related to the early identification—either through risk factors, biomarkers or self-reported symptoms—treatment interventions and service provision. All help to add to the evidence base for “*Neurological and clinical aspects of perinatal mental health*.” Many of these studies provide evidence for scalable real-world solutions and all identify future areas of research.

## Author contributions

All authors listed have made a substantial, direct, and intellectual contribution to the work and approved it for publication.

## References

[1] Howard LM Khalifeh H. Perinatal mental health: A review of progress and challenges. World Psychiatry. (2020) 19:313–27. 10.1002/wps.20769 32931106 PMC7491613

[2] Di Venanzio C Pacitti F Rossetti MC Santarelli V Gregori E D'Alfonso A. Perinatal depression screening and early treatment. J Psychopathol. (2017) 23:99–104.

[3] Hessami K Romanelli C Chiurazzi M Cozzolino M. COVID-19 pandemic and maternal mental health: A systematic review and meta-analysis. J Maternal Fetal Neonatal Med. (2022) 35:4014–21. 10.1080/14767058.2020.1843155 33135523

[4] Orsolini L Valchera A Vecchiotti R Tomasetti C Iasevoli F Fornaro M. Suicide during perinatal period: Epidemiology, risk factors, and clinical correlates. Front Psychiatry. (2016) 7:138. 10.3389/fpsyt.2016.00138 27570512 PMC4981602

[5] Gelaye B Rondon MB Araya R Williams MA. Epidemiology of maternal depression, risk factors, and child outcomes in low-income and middle-income countries. Lancet Psychiatry. (2016) 3:973–82. 10.1016/S2215-0366(16)30284-X 27650773 PMC5155709

[6] Maguire J McCormack C Mitchell A Monk C. Neurobiology of maternal mental illness. Handb Clin Neurol. (2020) 171:97–116. 10.1016/B978-0-444-64239-4.00005-9 32736761

[7] Somerville S Dedman K Hagan R Oxnam E Wettinger M Byrne S. The perinatal anxiety screening scale: Development and preliminary validation. Archiv Women's Mental Health. (2014) 17:443–54. 10.1007/s00737-014-0425-8 24699796

[8] Silverwood V Bullock L Turner K Chew-Graham C Kingstone T. The approach to managing perinatal anxiety: A mini-review. Front Psychiatry. (2022) 13:1022459. 10.3389/fpsyt.2022.1022459 36590629 PMC9797985

